# Emergence of *Francisella novicida* Bacteremia, Thailand

**DOI:** 10.3201/eid1412.080435

**Published:** 2008-12

**Authors:** Amornrut Leelaporn, Samaporn Yongyod, Sunee Limsrivanichakorn, Thitiya Yungyuen, Pattarachai Kiratisin

**Affiliations:** Siriraj Hospital, Mahidol University, Bangkok, Thailand

**Keywords:** Francisella novicida, bacteremia, Thailand, dispatch

## Abstract

We report isolation of *Francisella novicida*–causing bacteremia in a woman from Thailand who was receiving chemotherapy for ovarian cancer. The organism was isolated from blood cultures and identified by 16S rDNA and PPIase gene analyses. Diagnosis and treatment were delayed due to unawareness of the disease in this region.

*Francisella novicida*, a rare human pathogen, has recently been considered to be a subspecies of *F. tularensis* on the basis of DNA similarity (*1**,**2*). The reservoir and transmission route of *F. novicida* were not clearly defined. Since the first isolation of *F. novicida*, to our knowledge, only 5 patients with suspected infection have been reported (*3*–*5*). *F. novicida*, however, has neither been isolated nor associated with human disease in Thailand. We report a case of *F. novicida* infection in a Thai patient who was undergoing chemotherapy.

## The Study

In October 2007, a 37-year-old woman from Thailand sought treatment at Siriraj Hospital (a 2,400-bed university hospital in Bangkok, Thailand) with a history of fever for 1 week. She was a hairdresser residing in a suburban area of Prachuap Khiri Khan, a southern province of Thailand. She denied history of blood transfusion, animal contact, and travel abroad. She had not been aware of being bitten by insects recently. There was no incidence of unusual animal death in the area in which she resided. Five months before seeking treatment, she received a diagnosis of advanced stage clear cell adenocarcinoma of the ovary with metastasis to peritoneum, spleen, uterus, and multiple abdominal lymph nodes. Chemotherapy was planned. Initial laboratory screening showed increased liver enzyme levels and abnormal hepatitis markers confirming chronic active hepatitis B virus infection. Chemotherapy was delayed while she was treated with lamivudine. A follow-up visit in early September showed that her liver function biochemistry results had returned to within normal limits. Chemotherapy with carboplastin and paclitaxel was then initiated.

At the time of admission, 25 days after the start of chemotherapy, the patient had fever (39^o^C), blood pressure 90/60 mm Hg, and pulse rate 75 beats/min. She also had an episode of gastrointestinal hemorrhage with melena. It was believed that fever and gastrointestinal bleeding were complications from chemotherapy; thus, microbiologic investigation was not promptly initiated. Abnormal laboratory findings included anemia (hemoglobin 80 g/L) and leukocytosis with marked neutrophilia (Figure). Urine and stool cultures showed insignificant growth.

**Figure:**
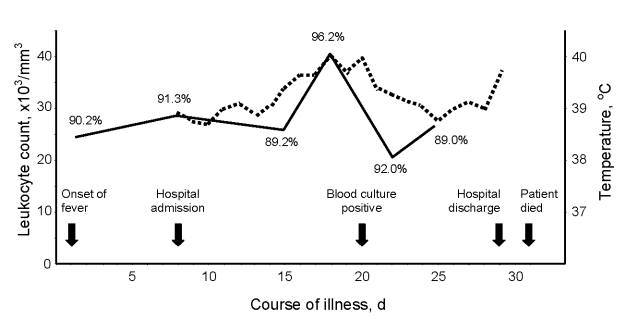
Course of illness in a Thai patient with *Francisella novicida* bacteremia. Solid line, leukocyte count with percentage of neutrophils; dashed line, temperature.

Two samples of blood cultures from peripheral lines were obtained using BacT/Alert FA bottles (bioMérieux, Durham, NC, USA) on day 10 of hospital admission and incubated in the continuous monitoring BacT/Alert 3D system (bioMérieux). Both blood culture bottles grew small pleomorphic gram-negative coccobacillus after incubation for 2 days. Samples from positive bottles were subcultured onto 5% (vol/vol) sheep blood agar, MacConkey agar, and chocolate agar. A slow-growing bacterium was recovered on both blood agar and chocolate agar after 2-day incubation at 35^o^C with 5% CO_2_. The organism was negative for catalase and oxidase. Bacterial identification was delayed because the organism was unidentifiable based on the conventional biochemical keys and the Vitek 2 system (bioMérieux, Marcy L’Etoile, France). Using the API 20NE (bioMérieux), the organism was identified as *Mannheimia haemolytica*/*Pasteurella trehalosi* with good confidence level (94.5% probability).

The identification of this isolate was further determined by the 16S rDNA gene analysis that used screening primers SQE1 and SQE3 (*6*) and the 1,445-bp gene sequencing procedure as described elsewhere (*7*). The DNA sequence was analyzed by using the standard nucleotide-nucleotide BLAST algorithm (www.ncbi.nlm.nih.gov)*.* The 16S rDNA sequence of this isolate, called strain RB401, was most closely related (99.9% homology) to the *novicida*-like subspecies of *F. novicida* strain 3523 (GenBank accession no. AY243028) and was deposited to the GenBank database under the accession no. EU365864.

Identification of strain RB401 was further confirmed by analyzing DNA sequences of TUL4 and PPIase genes as previously described for strain 3523 (*5*). The fragment containing TUL4 amplicon (363 nt) from strain RB401 shared the highest similarity (98%) to the matching region from strain 3523. Comparing the 267-nt coding region sequence of the 3′ end of PPIase gene nt 733259–733525 to the genome sequence of *F. novicida* strain U112 (GenBank accession no. CP000439) showed the highest similarity to all subspecies of *F. tularensis,* e.g., *F. tularensis* subsp. *tularensis* strain WY96-3418 (GenBank accession no. CP000608), and to *F. novicida* strain U112. The PPIase gene sequence of strain 3523 was not available for the alignment. However, the above findings also supported that our isolate was *F. novicida*. The sequences of TUL4 and PPIase genes from strain RB401 were deposited to GenBank under accession nos. EU786119 and EU786120, respectively.

Antimicrobial drug susceptibility testing was performed by disk diffusion method and interpreted based on the CLSI criteria for *Acinetobacter* spp. (*8*). Results showed that strain RB401 was susceptible to piperacillin/tazobactam, third-generation cephalosporins, cefepime, imipenem, meropenem, aminoglycosides, fluoroquinolones, and tetracycline, but resistant to co-trimoxazole. Due to the delay in organism identification and lack of awareness of its significance, antimicrobial treatment (piperacillin/tazobactam 4.5 g intravenously every 8 h) was not initiated until day 19. However, on day 21, the patient requested to be referred to her hometown hospital and died 2 days after referral. The final microbiology report was released 2 days after her death. The serologic test for antibodies against *Francisella* spp. was not available. All laboratory technicians who processed culture and identified the bacterium received doxycycline (100 mg twice a day for 14 days) for prophylaxis, and none reported fever or abnormal symptoms.

## Conclusions

*F. novicida*, often referred to as *F. tularensis* subsp. *novicida*, is rarely attributed to human infection and is not readily recognized in most clinical laboratories. Because of the unreliable results of phenotypic identification methods, unfamiliarity of the bacterium and close genetic relatedness among *Francisella* spp., the organism can be misidentified, thus leading to inappropriate management. A clinical diagnosis of *Francisella* infection is highly nonspecific, and it seems that the underlying disease with immunosuppression was an important factor to contracting the disease in the described case and previous case reports (*3**–**5*). Human *F. novicida* infection has not previously been described from Asia.

There is no clear explanation regarding route of acquisition and the pathogenic role of this organism. Additionally, there is no evidence for human-to-human transmission. In our case, clinicians and microbiologists did not suspect *F. novicida* infection. Given that our patient had severe complicated underlying diseases while the potentially low-virulence organism was recovered, it was thus difficult to claim that the patient’s death was solely due to *F. novicida* infection. Notably, the 16S rDNA, TUL4, and PPIase sequences of our isolate are most closely related to the strain earlier reported from Australia (*5*). These areas are not known to be endemic for *F. novicida,* and therefore, these strains represent the emergence of *F. novicida* in the Asia-Pacific region. The earlier case-patient was successfully treated with flucloxacillin and doxycycline, followed by dicloxacillin and doxycycline (*5*).

*F. novicida* is not considered to have a fastidious growth requirement. The standard protocol of 5-day incubation for automated blood culture (*9*) is supposedly sufficient for detection of *F. novicida* bacteremia. Identification of *F. novicida*, however, is often difficult because the bacterium can be easily misidentified as a non-*Francisella* species or as a highly pathogenic *F. tularensis*. It was also indicated that this organism could not be detected by a direct fluorescent antibody test used for the identification of *F. tularensis* types A and B (*5*). As a preliminary result, our 16S rDNA sequence was closely related to subspecies of *F. tularensis*, and thus doxycycline prophylaxis for the organism-exposed laboratory personnel could not be avoided. There is high risk for laboratory-acquired tularemia when handling *F. tularensis* cultures (*10*).

In retrospect, prophylactic treatment may have been unnecessary because our isolate proved to be *F. novicida,* which is not known to cause laboratory-acquired infections. Rapid methods for *Francisella* species identification are needed for better consideration of antimicrobial prophylaxis. Most *F. tularensis* strains were susceptible to aminoglycosides, quinolones, and tetracyclines (*11*). Our isolate was similarly susceptible to these agents, which suggests that they may be considered as therapeutic options for *Francisella* spp. Initial treatment with piperacillin/tazobactam was based on the preliminary report of gram-negative bacilli, but later antimicrobial susceptibility testing showed this isolate to be susceptible to this agent. However, previous reports (*11**–**13*) suggested that piperacillin/tazobactam and other β-lactams are generally considered ineffective in vivo against *Francisella* spp. and likely result in therapeutic failure. Given that the organism is believed to be of low virulence, particularly that it does not pose a substantial risk for immunocompetent persons, its role as a human pathogen remains controversial. Further studies to gain better understanding of pathogenic mechanism and transmission route of *F. novicida* are necessary to provide appropriate guidelines for the treatment and prophylaxis.
